# Adjustable Versus Nonadjustable Sutures in Strabismus Surgery—Who Benefits the Most?

**DOI:** 10.3390/jcm9020292

**Published:** 2020-01-21

**Authors:** Maciej Gawęcki

**Affiliations:** Dobry Wzrok Ophthalmological Clinic, Kliniczna 1B/2, 80-402 Gdansk, Poland; gawecki@use.pl; Tel.: +48-501-788654

**Keywords:** adjustable sutures, strabismus surgery, paretic strabismus

## Abstract

Background: Adjustable sutures have been used in strabismus surgery for more than 40 years, but controversy remains regarding their application. This review sought to analyze studies comparing the efficacy of adjustable sutures (AS) and nonadjustable sutures (NAS) in the treatment of different ocular deviations. Materials and Methods: The PubMed literature database was searched using the keywords ‘adjustable sutures’ and ‘strabismus surgery’, yielding a total of 209 results. Only comparative studies were extracted, and the results were divided into three categories: Adult comitant strabismus, childhood comitant strabismus, and paretic/restrictive strabismus. Results: The search revealed eleven comparative studies on AS versus NAS in adult strabismus, including only one randomized controlled trial. Five of these studies analyzed just the postoperative success rate, three studies analyzed just the reoperation rate, two studies analyzed both the postoperative success and reoperation rates, and one study evaluated achievement of the postoperative target angle. Three of seven studies analyzing postoperative success reported the statically significant superiority of AS over NAS, while four of five studies analyzing reoperation rate indicated a significantly smaller percentage of reoperations with the use of AS. The study covering postoperative target angle as an outcome favored the AS technique. Separately, the search revealed three comparative studies on AS versus NAS in childhood strabismus, one of which reported a statistically significant advantage with AS. Only four comparative studies on AS versus NAS in paretic or restrictive strabismus were found; all showed a tendency for better results with the use of AS but not in a statistically significant fashion. Overall, out of 18 studies analyzed in this review, 17 suggested better clinical results followed the application of AS versus NAS; however, only a minority had statistically significant results. Conclusion: The analysis of available research failed to support AS as the preferable surgery technique over NAS in cases of simple and predictive strabismus. Further research is needed to more precisely determine the group of patients able to benefit the most from AS.

## 1. Introduction

Adjustable sutures (AS) have been used in strabismus surgery for a few decades since their first description by Jampolsky in 1974; however, controversy remains regarding their application in clinical practice [1]. Many surgeons advocate for AS during surgery for the management of most cases of strabismus, while others limit their use to when treating adults or in selected complicated cases such as paralytic strabismus, restrictive strabismus, and reoperations [2].The question of careful selection of patients suitable for AS is often brought up [3]. AS surgery is reported to be used less frequently than classic nonadjustable sutures (NAS). According to the Intelligent Research in Sight registry of the American Academy of Ophthalmology, AS were used in 7.42% of strabismus surgery cases [4]. Some clinicians do not see the advantage of adopting AS over NAS in every patient, especially in cases of simple comitant strabismus. They also point out that the adjustment procedure is uncomfortable and stressful for the patient and requires special nursing care [5]. Interestingly, Vassconcelos and Guyton in their editorial for *Arquivos Brasilerios de Oftalmologia* indicated that the adoption of AS in strabismus surgery is partly related to surgical experience [6]. Successful use of AS requires a long apprenticeship and often promotes a high load of stress at the beginning of the learning curve, so some surgeons favor the simpler NAS technique. 

This study sought to review the medical literature covering the results of both techniques to elucidate the group of patients who would benefit the most from the AS technique. This paper specifically concentrated on studies that presented results of AS versus NAS surgery in a comparative fashion. 

## 2. Materials and Methods

The PubMed literature database was searched according to the keywords ‘adjustable sutures’ and ‘strabismus surgery’. A total of 209 results were found, from which only comparative studies were extracted. Results of the search were divided into three groups according to subject matter: Comitant deviation in adults, comitant deviation in children, and paralytic or restrictive strabismus. 

Ethical statement: The study had a character of a review, so formal consent for its conducting was not needed.

## 3. Results

Of the 18 studies ultimately selected for inclusion in this review, the database search revealed just two randomized controlled trials (RCT) comparing the results of AS versus NAS [7,8]. The first study included 60 children with an average age of 3.5 randomized into AS and NAS groups, respectively. Adjustment was performed usually a few hours after surgery under propofol. Success was defined as a final deviation of 8 prism diopters or less of straight at six months and was achieved in 88.67% of the AS group versus 73.33% of the NAS group. The authors thus reported the results favored AS as clinically preferable, although the difference between the techniques was not statistically significant. 

The second study involved 40 adults with intermittent exotropia. Similarly, there was no significant difference found between AS versus NAS regarding the postoperative success rate (90% for AS vs. 85% for NAS; *p* = 0.3). The details of these studies are presented in Table 1 and Table 2.

Altogether, we identified 11 comparative studies that reported on AS versus NAS in adult strabismus surgery, including one of the RCTs mentioned above. Five of these studies analyzed just the postoperative success rate, three studies analyzed just the reoperation rate, two studies analyzed both the postoperative success and reoperation rates, and one study evaluated the achievement of the postoperative target angle. The results of these studies are presented in Table 1. 

### 3.1. Postoperative Success Rate

Seven studies altogether analyzed the postoperative success rate in AS versus NAS, usually at three to six months after surgery. 

Only three studies suggested statistically better results of surgery were achieved with the use of AS [9,10,11]. The remaining four studies did not prove the statistical superiority of either of the two surgical methods over the other [8,12,13,14]. 

One study concentrated on achievement of the target postoperative angle and, in that respect, strongly favored AS over NAS for both horizontal and vertical deviations [15]. 

### 3.2. Reoperation Rate

Three studies concentrated solely on evaluating the reoperation rate in AS versus NAS [16,17,18] and two analyzed both the postoperative success rate and the reoperation rate [10,11]. Two of these studies, both by Leffer and colleagues, suggested a lower reoperation rate was achievable with the use of AS in horizontal strabismus but not vertical strabismus [16,17]. Studies by Tripathi et al. [18] and Zhang et al. [11] favored AS independently of deviation type, while the study by Mireskandari et al. [10] did not report a statistically significant difference in reoperation rate between the AS and NAS techniques.

Only one study—on sensory exotropia—showed better clinical results were attainable using NAS, without statistical significance. All other included studies reported clinically better results were obtained with the use of AS, although this result was not backed by statistical significance either.

Separately, the review of the medical literature revealed just three comparative studies on AS versus NAS in children. The results of the search in this regard are presented in Table 1. 

A large study by Kassem et al. (*n* = 637 patients) showed the superiority of AS over NAS in achieving the expected postoperative deviation [19]. On the other hand, the aforementioned RCT by Kamal et al. did not prove that effect, although their sample population was much smaller (*n* = 60 patients) [7]. A study by Leffer et al. concentrated on the reoperation rate in AS and NAS but did not reveal any statistically significant difference between the two groups [20]. Again, as seen among adults, the latter two studies clinically favored the AS technique, but without statistical significance. 

There were very few studies available that compared the efficacy of AS versus NAS in paralytic and restrictive strabismus. The results of the search in this regard are presented in Table 3.

The available research does not favor AS if statistical significance is the only criterion; however, some clinical results suggest a tendency for better postoperative outcomes with the use of AS [2,21]. Nevertheless, the possibility of substantial postoperative drift must be taken into consideration when using AS in vertical strabismus [22]. 

## 4. Discussion

The present analysis of available studies comparing the AS and NAS techniques does not yield statistically significant results that unequivocally favor the use of AS (or NAS) in any form of strabismus. Most of the studies that analyzed the reoperation rate in AS versus NAS showed, however, that the percentage of patients who require a second surgery is significantly lower following the use of AS. 

The author believes that the interpretation of the results of clinical research on the subject of AS versus NAS must go beyond just statistics. Concentrating solely on RCTs will not provide any conclusions, as so far only two such studies were performed [7,8]. Authors of recent Cochrane Database Systemic Reviews on AS versus NAS in strabismus surgery were not able to present any reliable thesis due to the scarceness of randomized studies on the subject [24]. 

Many authors choose AS as their preferred procedure in treating complicated forms of strabismus, and thus do not perform any comparative studies with NAS. Besides, it should be emphasized that, generally, rates of postoperative success are high for both AS and NAS; thus, the statistical superiority of either of these two procedures over the other is hard to prove. 

Almost all of the studies included (besides one on sensory exotropia) suggest the clinical superiority of AS versus NAS but not in a manner that reached statistical significance. This fact should not be ignored and could suggest that AS works better for certain groups of patients but not for all patients. It has to be taken into consideration that some deviations drift with time, no matter which surgical technique is used. As we see from the study by Mireskandari et al., AS is significantly better in achieving immediate postoperative success, but this was not necessarily a long-term effect [15]. With the more precise selection of patients for AS surgery, the statistical significance in many studies could more likely have been achieved. 

It has to be said, that only a percentage of patients operated on with AS require adjustment. In the study by Weston et al. (*n* = 201 consecutive patients), just 40% of cases required suture adjustment, while the remaining 60% were simply tied off [25]. Agnello et al. reported a necessity for adjustment in 62.3% of their AS strabismus surgeries (48/77 patients) [26]. Pratt-Johnson et al. reported 48.2% (123/255) of patients required adjustment in AS surgery cases [27]. However, this number was as low as 26% in the study of McNeer et al. (vertical strabismus) [28].

These data suggest that, on average, half of normal strabological measurements work well without the necessity for adjustment. In this context, research should focus on determining the patients who would benefit most from AS strabismus surgery without the necessity to adjust all of them. Considering AS to be a superior technique to NAS in treating simple forms of strabismus is questionable.

Most of the papers available in PubMed database are case series presenting optimistic results of AS surgery but not really comparing the results of strabismus surgery between AS and NAS [29,30]. Often, authors point out the beneficial role of AS in complicated cases of strabismus and the reduced need for reoperation with the use of the technique [31,32].

It should be emphasized that experienced ophthalmic surgeons who actually use AS are eager supporters of the technique and often tend to abandon NAS in most adult cases of strabismus. This explains why comparative studies on AS versus NAS are so sparse. However, followers of AS defend its superiority over classical strabismus surgery based on personal experience and clinical practice but not necessary RCTs. 

The act of adjustment itself creates most of the controversies and arguments. Adversaries to AS usually bring up the patient’s discomfort as a major problem seen during the AS procedure. Some clinicians believe that patients should be carefully selected according to their potential cooperation for the adjustment procedure [3,33]. Therefore, they exclude children as potential candidates. Adjustment is sometimes treated as an additional surgery that involves significant stress and often vagal responses during adjustment (e.g., nausea and vomiting) [34,35]. On the other hand, it is possible to perform adjustment as a one-stage procedure just after the main surgery, when the anesthetics are still working. Karaba et al. suggested the one-stage surgery technique in their report as being less stressful for the patient [36]. The same conclusions were provided by Cogen et al. [37]. Other authors provide modifications of AS technique to reduce the burden of adjustment. Nihalani et al. proposed short tag noose technique, which avoids the need for sedation in patients who do not require adjustment [38]. Robbins et al. described similar closed conjunctival delayed adjustable technique [39], and Budning et al. also similar short adjustable suture technique [40].

The advantages of AS use versus the relative potential discomfort of adjustment and the burden of reorganizing the operating theatre have to be balanced, and those patients who would benefit more from AS than NAS should be carefully selected.

The use of adjustable sutures in children also needs more comparative research. Ophthalmological centers that apply AS in children report good postoperative success rates. Again, these are mainly reports of postoperative outcomes rather than comparative studies [41,42]. In our analysis, one study strongly favored the use of AS in children, while another two papers did not report a statistically significant superiority of AS over NAS; however, the clinical tendency is to see better results with AS than NAS.

It is well-known from ophthalmological practice that not many surgeons routinely use the AS technique in children. Minors are usually less cooperative than adults, so performing the adjustment requires additional anesthesia, usually intravenous propofol or sometimes intranasal midazolam [43]. That is why, for some clinicians the whole procedure seems troublesome, and very young patients are considered not suitable for adjustment. However, this might be mainly due to a lack of experience with applying anesthesia in children, the belief that the procedure must be painful, and that successful outcomes require strict cooperation. Meanwhile, some surgeons perform use a single-stage adjustment surgery approach also in children. The use of general anesthesia with immediate postoperative adjustment requires a specific anesthetic technique, though. Usually, intravenous propofol is titrated in such situations [27,44]. Stopping propofol infusion enables the surgeon to perform the adjustment within minutes while the anesthetics are still working. 

Without a doubt, the application of AS in children requires a certain organization of the operation theatre and involvement of a skillful anesthetic team. Probably, this is why it is not commonly applied in treating childhood strabismus. However, theoretically speaking, proper ocular alignment at a young age is expected to be beneficial for the development of the visual system and binocularity. Logically, the use of AS in children should provide better functional effects than that in adults. Many studies in the literature support benefits of early ocular alignment [45,46,47,48], however, the optimal age for strabismus surgery is still debatable [49,50].

The analysis of comparative studies on AS versus NAS in cases of paretic and restrictive strabismus did not prove a statistically significant advantage inherent with the use of AS; however, again AS showed a clinical tendency to lead to better results. Usually, studies include a small number of cases, so it is difficult to conduct a proper statistical analysis. Besides, as paretic or restrictive strabismus is difficult to treat, very often, AS is used as a method of choice without involving a control group composed of NAS procedures.

Nevertheless, the use of AS in treating vertical deviations, especially in thyroid ophthalmopathy (TO), is controversial and, so far, there is no consensus regarding the use of AS in these disorders. One of the studies included in this review reported significant postoperative drift occurred in cases of vertical strabismus treated with AS [22]. Still, some surgeons advocate for the use of AS in the inferior rectus (IR) recession. Volpa et al. reported a large case series of 54 patients treated with AS due to vertical strabismus secondary to TO [51]. At a mean follow-up of 38 weeks, 35 patients (65%) presented excellent results. Over-correction occurred in just 20% of cases (*n* = 11 patients). On the other hand, Kerr et al. revealed that over-correction in IR recession by AS is strongly correlated with TO and, thus, NAS should be used in such cases [52]. The same conclusion was provided by Shokida et al. and Schittkowski et al. [53,54]. Kushner suggests an interesting alternative for IR fixed recessions to avoid over-corrections involving applying semi-adjustable suture in such cases [55]. During this approach, the corners of the muscle are sutured firmly to the sclera, but its central part is placed using AS. Vazques et al. recommend pursuing under-correction when adjusting sutures in IR recessions [56].

In clinical practice, many surgeons use adjustable sutures as their method of choice for complicated cases of strabismus, such as paretic and restrictive strabismus, strabismus with unstable angle, or strabismus in high myopia [57,58,59,60]. A good example for that is the analysis of strabismus surgeries (2000–2008) performed at Moorfields Eye Hospital in patients older than 60 years of age indicated the use of AS occurred in 61% cases (*n* = 144 patients) [61]. Major causes for surgery in this group included TO, sixth nerve palsy, and iatrogenic causes, but not comitant strabismus as seen in young individuals. Similar indications for strabismus surgery in patients over 60 years of age have been listed by Magramm et al. [62]. Paralytic and restrictive strabismus are common indications for the use of AS [63,64]. The AS technique can also be applied with success during superior oblique tendon surgery [65]. Some surgeons willingly use AS in cases of large-angle strabismus, when surgical tables are often misleading [66]. AS has been reported as an effective approach in cases of incomitant strabismus [67]. 

Available data prove that the AS technique in the hands of an experienced surgeon is a generally safe and comfortable procedure. Most of the operated patients do not complain of serious pain or discomfort during adjustment. A large study conducted in Germany questioned 113 patients who underwent the adjustment procedure more than 10 years before the survey and 89.4% of patients either did not remember the adjustment or did not have any complaints about the procedure [68]. 

The AS procedure does not affect conjunctival healing significantly. Studies have suggested that eye redness post-surgery not persist significantly longer in eyes operated on with this technique relative to those who underwent NAS [69].

The AS technique, however, does requires some special attention. There exists a potential for drift toward under-correction with the use of this procedure so, generally, the adjustment should include some over-correction [70]. Some studies have supported, however, that the drift seen in AS is not larger than that which occurs with NAS [30].

Importantly, the present review does not support the superiority of AS over NAS in the majority of strabismus patients; however, the author hopes not to discourage ophthalmologists from using AS. On the contrary, the author supports gathering further results from everyday clinical practice and rationale to support the use of AS in complicated forms of strabismus, especially reoperations and unpredictable cases. More research and opinion-sharing is needed in the treatment of childhood strabismus with AS, as the author believes that increasing the frequency of use of this technique in minors is a technical concern rather than a substantial one. 

## 5. Conclusions

The analysis of available research did not provide an univocal answer to support that AS is superior to NAS in simple comitant strabismus. More precise selection of patients for the AS surgery could provide the data on benefits of this form of surgery. Research aimed at determining the optimal group(s) of patients with strabismus who would benefit most from AS over NAS should be carried out. The use of AS in children requires the specific organization of the operating theatre and anesthetic team. Further research is needed to confirm adequate long-term functional benefits of AS use in children. 

## Figures and Tables

**Table 1:** Studies comparing the effects of treatment according to AS versus NAS.

Author	Material and Study Design	Type of Deviation Analyzed	Results	Statistical Significance
Babu et al. [3].	Randomized, prospective, interventional 40 adults with intermittent exotropia randomized either to AS (20 eyes) or NAS (20 eyes) technique for bilateral LR recession.	Intermittent exotropia.	Success defined as postoperative deviation <10 PD Evaluation at 12 weeks: Success in 90% of patients in AS group and 85% in NAS group. Mean residual deviation in AS group: 6.2 PD, in NAS group: 5.6 PD.	*p* = 0.316; difference insignificant.
Ferdi A et al. [12]	27 consecutive patients operated at one hospital; 11 AS, 16 NAS. Out of 11 AS patients, only 6 had adjustment made.	Horizontal.	Mean postoperative deviation: 11.6 PD in AS group and 15.3 PD in NAS group.	Postoperative deviation: *p* = 0.519, difference insignificant.
Leffler CT et al. [16]	Reoperation rate in AS and NAS group during one calendar year. Analysis of fee-for-service payments to Medicare in 2012.	Horizontal and vertical.	Reoperation rate for horizontal strabismus: 4.1% (15/364) for AS and 7.1% (77/1,082) for NAS. In vertical strabismus: 4.1% (8/196) for AS and 8.3% (38/458) for NAS.	*p* = 0.047 for horizontal strabismus and *p* = 0.07 for vertical strabismus. Difference significant for horizontal strabismus.
Agrawal et al. [9]	Prospective, comparative, nonrandomized study. 54 patients with horizontal strabismus operated with AS (27) or NAS (27).	Horizontal.	Success defined as postoperative deviation within ±10 PD at 6 months. Success rate: 88.8% (24 patients) in AS group and 62.9% (17 patients) in NAS group.	*p* = 0.02, statistically significant.
Leffler et al. [17]	Retrospective, review of insurance database 2007–2011. Reoperation rate among 6178 strabismus surgeries in adults according to AS or NAS technique.	Horizontal and vertical.	Total reoperation rate: 8.5% AS group: 8.1% vs. NAS group: 8.6%. For horizontal deviations, reoperation rate 5.8% in AS group and 7.8% in NAS group. For vertical deviations, reoperation rate 15.2% in AS group and 10.4% in NAS group.	For total reoperation rate: *p* = 0.57, difference insignificant. For horizontal strabismus: *p* = 0.02, difference significant. For vertical deviations: *p* = 0.05, borders on statistical significance.
Mireskandari et al. [15]	Retrospective. 353 patients older than 12 years. Chances of achieving target postoperative deviation with AS versus NAS. Target deviation: for esotropia and vertical, within 4 PD; for exotropia, between orthotropia and 8 PD esotropia	Horizontal and vertical.	AS group achieved target angle in 75.5% cases versus 54% in NAS group.	*p* < 0.0001, statistically significant.
Liebermann et al. [13]	Retrospective. 89 consecutive patients operated with AS 60% (53) adjusted. 40% (36) tied off (good effect during adjustment).	Horizontal, reoperation.	Long-term results (success if deviation <10 PD and no diplopia): Success rate at 6 weeks: 64% for not adjusted and 81% for adjusted. At 1-year success rate: 67% for not adjusted and 77% for adjusted.	*p* = 0.09 for 6 weeks and *p* = 0.3 for 1 year. Difference not significant.
Mireskandari et al. [10]	Retrospective, data from 13 years, one surgeon. 144 patients; primary operations and reoperations.	Horizontal and vertical.	Success defined as postoperative deviation within 10 PD for horizontal and 5 PD for vertical strabismus, lack of diplopia. Success rate: 77.7% for AS and 65.9% for NAS. For exotropia, success rate: AS 80.8% for AS versus 65.9% for NAS. For primary surgery success rate: 82.5% for AS versus 50% for NAS. Reoperation: 80.2% for AS and 77.6% for NAS.	For overall: *p* = 0.059, not significant. For exotropia: *p* = 0.011, significant. For primary surgery: *p* = 0.003, significant. For reoperations: *p* = 0.71, not significant.
Zhang et al. [11]	Retrospective, data from 1989–2013. 535 consecutive strabismus patients; included 491 patients: 305 AS and 186 NAS.	Horizontal and vertical.	Success: postoperative deviation ≤10 PD horizontal and ≤2 PD vertical. Follow-up: 7 days to 12 weeks. Overall success rate: 74.8% for AS and 61.3% for NAS. Reoperations success rate: 65.7% for AS versus 42.4% for NAS. Childhood strabismus primary success rate: 81.4% for AS versus 65% for NAS. TO success rate: 74.1% for AS versus 76.7% for NAS.	Overall: *p* = 0.0016, significant. Reoperations: *p* = 0.03, significant. Childhood primary: *p* = 0.135, insignificant. TO *p* = 0.82, insignificant.
Park et al. [14]	Retrospective, data from 1998–2005 54 patients with sensory exotropia; 34 operated with NAS, 20 operated with AS on LR.	Sensory exotropia.	Postoperative success defined as deviation <15 PD at 3 months. Success: 75% (15 patients) in AS group versus 88% (30 patients) in NAS group.	*p* = 0.944, not significant.
Tripathi et al. [18]	Retrospective, data from 1996–2000. 443 patients older than 13 years. 141 operated with AS, 302 operated with NAS. Analysis of reoperation rate.	All types.	Reoperation rate: 8.51% for AS and 27.15% for NAS.	*p* < 0.005, significant.

AS: adjustable sutures; NAS: nonadjustable sutures; PD: prism diopters; LR: lateral rectus; IR: inferior rectus; TO: thyroid ophthalmopathy.

**Table 2:** AS versus NAS in children.

Author	Study Design and Material	Deviation Type	Type of Anesthesia	Results	Statistical Significance
Kassem et al. [2]	Retrospective analysis of records of consecutive patients aged ≤15 years operated on from 1989 through 2012. Material: 521 patients in AS group and 116 patients in NAS group.	Horizontal.	Adjustment in proparacaine (15%) and intravenous propofol (85%).	Success defined as deviation within 8PD of straight at 3–6 months: 77.7 % in AS group 64,6% in NAS group.	*p* ≤ 0.03; significant.
Kamal et al. [2].	RCT of AS versus NAS in children. Material: 30 eyes operated by AS, mean age 3.48 ± 2.37 and 30 eyes operated by NAS 3.55 ± 2.64.	Horizontal.	Adjustment under Propofol; mean 156.5 ± 46.6 min. after the main surgery.	Success defined as final deviation ≤8 PD of straight at 6 months: 88.67% in AS group 73.33% in NAS. Over-correction 13.33% in AS group versus 3.33% in NAS group. Under-correction 0% in AS versus 23.33% in NAS group.	*p* = 0.197; statistically insignificant.
Leffler et al. [20]	Retrospective. Analysis of insurance database 2007–2013; reoperation rate in AS and NAS technique. Material: 11,115 documented strabismus operations in patients under 18 years of age.	All types.	Not analyzed.	Total reoperation rate: 7.7%. Specifically, in AS group: 7.4%, in NAS group: 9.6%.	*p* = 0.18; difference statistically insignificant.

AS: adjustable sutures; NAS: nonadjustable sutures; PD: prism diopters; RCT: randomized controlled trial.

**Table 3:** Paralytic or restrictive strabismus surgery.

Author	Material and Study Design	Type of Deviation Analyzed	Results	Statistical Significance
Peragallo et al. [2]	Retrospective. Material: 56 patients with III nerve palsy. AS in 27 patients (48%), NAS in 29 (52%).	IIIrd nerve palsy.	Success: postoperative deviation ≤10 PD horizontal and ≤2 PD vertical. 63% for AS and 38% for NAS.	*p* = 0.06, difference insignificant.
Peragallo et al. [22]	Retrospective, controlled. Evaluation of IR recession and postoperative drift in TO with the use of AS versus NAS and confronted with control group patients who underwent IR recession due to other disorders than TO.	TO, hypotropia.	AS group: before surgery: 17 PD ±9; Day 1: 1.2 ± 2.5; final: −0.7 ± 5.6 NAS group before surgery: 21 ± 7; Day 1: 3.7 ± 4.9; final: 2.7 ± 6.7 Controls: before surgery 11 ± 4; Day 1: 0.3 ± 2.4; final 1.7 ± 5.7 Drift values: −1.9 ± 4.3 for AS; −1.0 ± 4.6 for NAS; 1.4 ± 5.9 for controls.	*p* = 0.05 for the drift between AS group versus controls.
Karhanova et al. [21]	Retrospective. 14 patients with TO operated on with AS (*n* = 7) or NAS (*n* = 7) technique.	TO, restrictive.	AS: no need for reoperation or prismatic correction. NAS: 2 patients required reoperation and 2 required prismatic correction.	Sample too small to show statistical significance.
Kraus et al. [23]	Retrospective. 37 patients who underwent IR recession, 26 NAS and 11 AS.	TO.	Success defined as fusion in primary and reading position without prisms. Results: AS: success in 64% (7/11) without prism, 91% (10/11) with or without prisms. NAS: success in 38% (10/26) without prism and 65% (17/26) with or without prism. Reoperation rate: AS: 9% (1/11) NAS: 35% (9/26)	*p* = 0.279 for success; *p* = 0.224 for reoperation rate; Not significant.

AS: adjustable sutures; NAS: nonadjustable sutures; PD: prism diopters; IR: inferior rectus; TO: thyroid ophthalmopathy.
